# Detection of Human Papillomaviruses in the Nasopharynx of Breastfed Infants: New Findings and Meta-Analysis

**DOI:** 10.3390/v12101119

**Published:** 2020-10-01

**Authors:** Luisa Dassi, Clorinda Annunziata, Chiara Botti, Alberto Micillo, Andrea Cerasuolo, Noemy Starita, Franco M. Buonaguro, Maria Lina Tornesello

**Affiliations:** 1 Molecular Biology and Viral Oncology Unit, Istituto Nazionale Tumori IRCCS-Fondazione G. Pascale, 80131 Napoli, Italy; luisa.dassi@istitutotumori.na.it (L.D.); c.annunziata@istitutotumori.na.it (C.A.); a.cerasuolo@istitutotumori.na.it (A.C.); n.starita@istitutotumori.na.it (N.S.); f.buonaguro@istitutotumori.na.it (F.M.B.); 2 Laboratorio di Patologia Clinica, Ospedale Santobono, 80131 Napoli, Italy; chiara_botti@yahoo.it (C.B.); a.micillo@santobonopausilipon.it (A.M.)

**Keywords:** human papillomavirus (HPV), alpha HPVs, beta HPVs, breastfeeding, infants

## Abstract

Vertical transmission of human papillomaviruses (HPVs) from mother to infant is known to occur during labor, delivery or breastfeeding. Infection with mucosal HPV 6 and 11 may cause recurrent respiratory papillomatosis in children, which is a rare and severe respiratory disease. The cutaneous HPV genotypes have also been described to be transmitted from mother to newborn through skin-to-skin contacts and during breastfeeding. To investigate the perinatal transmission of alpha and beta HPVs we collected nasopharyngeal specimens from 0–12-months-old infants born by vaginal delivery and breastfed at the time of sample collection. The mucosal and cutaneous HPVs were searched by nested PCR using the MY09/11-MGPs and CP65/70-CP66/69 primer sets, respectively, and genotypes identified by direct sequencing analysis. Fourteen out of 113 (12.4%) samples tested positive for HPV and sequence analysis allowed us to identify eight beta genotypes (HPV 5b, 20, 25, 100, 107, 124, 152 and RTRX7). Moreover, we performed a comprehensive review of published studies on the prevalence of mucosal and cutaneous HPVs among 5126 newborns and observed that 10% and 53% were positive for alpha and beta HPVs, respectively. In all studies there was an inverse correlation between the rate of alpha HPV positivity and age, while a significant positive trend was observed in beta HPV detection and age with the highest rate among children older than 12 months (Χ^2^ test for trend of 10.6, *p* < 0.001). Further studies are needed to confirm the hypothesis that beta HPVs are transmitted to breastfeeding infants through shedding of viruses in the breast milk or on the external breast epithelium.

## 1. Introduction

More than 200 human papillomaviruses (HPVs) have been fully characterized and new genotypes are often identified in metagenomic libraries obtained by the application of highly sensitive techniques [1,2]. Depending on their nucleotide sequence homology the HPVs are classified into five genera, defined as alpha (α), beta (β), gamma (ɣ), mu (μ) and nu (ν) [3]. HPV genotypes of different genera have a sequence similarity below 60% while those within the same genus have a homology between 60% and 70% in their L1 gene [4]. High risk alpha HPVs, particularly genotypes 16 and 18, are associated with cancers arising at mucosal sites such as the upper aero-digestive tract and ano-genital tract [5]. Low risk alpha genotypes, mainly HPV 6 and 11, cause benign hyper-proliferative lesions such as respiratory tract papillomatosis and genital warts [6,7,8].

HPVs have a circular double-strand DNA genome of approximately 8000 base pairs that is divided into three major regions: (1) the “upstream regulatory region” (URR), also named “long control region” (LCR), which contains the enhancer and other regulatory elements; (2) the early gene region containing the E1–E8 Open Reading Frames (ORFs) that encode proteins involved in viral genome maintenance and replication; and (3) the late gene region that contains the L1 and L2 ORFs expressing the major and minor capsid proteins, respectively. The transforming ability of high risk alpha HPVs is mainly due to the constitutive expression of E5, E6, and E7 oncoproteins that interact with multiple cell factors, such as p53 and pRb oncosuppressors, thus causing uncontrolled cell replication [9]. The role of beta HPVs in carcinogenesis is controversial. The early genes E6 and E7 encoded by beta HPVs have been found expressed in a subset of skin squamous cell carcinoma. Moreover, in vitro studies showed that these E6 and E7 oncoproteins are able to promote proliferation and to overcome cellular stresses induced by UV radiation [10].

Transmission of HPVs from mother to child has been reported by several studies. The virus may be transferred during pregnancy, through transplacental or perinatal transmission, or by nursing after delivery [11,12,13]. Indeed, both high and low risk HPVs have been found to cross the placenta and to spread to the child with a detection rate varying from 0% to 42.5% [11]. Maternal immune deregulation during pregnancy has been described to be associated with increased risk of viral transmission to the fetus [14,15]. Rombaldi et al. reported that HPV was mainly detected between the first and the sixth month of life with a high concordance rate of viral genotypes between children and mothers proving the vertical transmission [16]. The HPVs most frequently detected in the newborns, in this study, were genotypes 6/11 (53.3%), 42, 18, and 52 (13.3% each) as well as 59 (6.7%) [16]. The presence of anogenital warts in pregnant women, commonly caused by low risk HPV types (6, 11, 40, 42, 43, 44, 53, 54, 61, 72, 73 and 81), is associated with an increased incidence of Juvenile Recurrent Respiratory Papillomatosis (JoRRP) in their children [17]. The earlier the onset of JoRRP, the more aggressive the disease will be. In the United States the incidence of JoRRP is estimated to be 4.3 per 100,000 children and the infection with HPV 6 and 11 remains the main cause [18,19,20]. On the other hand, high risk HPVs, such as type 16, have been also detected in the oral mucosa and buccal swabs of children that are up to 12 years old [21]. 

It has been suggested that HPV can be transferred vertically by breastfeeding [22]. De Villiers et al. have shown that HPV prevalence is very high (86%) in the nipple and areola epithelia of breast cancer patients [23]. The HPV genotypes 6 and 11 were the most prevalent both in breast carcinoma biopsies (69%) and in the mamilla control samples (41%). Moreover, the HPV 27, frequently associated with cutaneous warts, and the HPV 57, identified either in mucosal or cutaneous lesions, were also frequently detected in breast carcinoma and control tissues [23]. It has been hypothesized that HPVs could spread from nipple and areola to the lactiferous ducts and sinuses and, via epithelial microlesions, could infect basal cells and start replication by expressing early genes E5, E6, E7, E1 and E2 [1,24,25,26].

The studies on HPV vertical transmission are still controversial and scant. Our study aimed to analyze the mucosal and cutaneous HPV prevalence in nasopharyngeal samples obtained from children up to 1 year old who were born by vaginal delivery and breastfed. We also performed a meta-analysis of published studies to investigate the overall prevalence of alpha and beta HPV infection in nasopharynx of the children. 

## 2. Materials and Methods

### 2.1. Subjects and Biological Samples

Nasopharyngeal aspirates were collected from 113 breastfeeding infants, aged between 12 days and 12 months of life, seeking care for respiratory problems at the Santobono hospital in Naples from September 2018 to January 2019. All children included in the study were born by vaginal delivery. The study was designed as a retrospective analysis, it is in accordance with the principles of the Declaration of Helsinki and was approved by the Institutional Scientific Board of the Istituto Nazionale Tumori IRCCS “Fondazione G. Pascale”.

### 2.2. DNA Extraction

Genomic DNA was extracted from nasopharyngeal specimens by digestion with proteinase K (150 μg per mL at 37 °C for 30 min) in 100 μL of lysis buffer (10 mM Tris–HCl pH 7.6, 5 mM EDTA, 150 mM NaCl, 1% SDS), followed by extraction with phenol–chloroform–isoamyl alcohol (25:24:1) and ethanol precipitation in 0.3 M sodium acetate (pH 4.6). The quality and quantity of isolated nucleic acid was spectrophotometrically assessed with Nanodrop 2000C (Thermo Fisher Scientific, Waltham, MA, USA). All samples with a ratio of absorbance at 260 and 280 nm equal or above 1.8 were included for further analyses.

### 2.3. HPV Detection and Genotyping

Nucleic acid integrity was assessed by PCR amplification of a 150 bp fragment within the exon 7 of TP53 gene (Table S1) [27]. HPV detection was carried out by nested PCR [28], as shown in Table S1, using the following primer pairs: (a) MY09/MY11 consensus primers [29] followed by the MGPs primer system for the amplification of mucosal HPV [30]; (b) CP65/CP70 followed by CP66/CP69 for the amplification of *Epidermodysplasia Verruciformis* (EV)-related HPV types [31]. A negative control sample, made of a reaction mixture without template DNA, was included in every set of five clinical specimens for each PCR run. Serial dilutions (from 1 to 1000) of plasmid clones containing mucosal (HPV 16) and cutaneous (HPV 5, 8 and 10) viral genomes were used as positive controls. The PCR system used for alpha HPV detection was evaluated for its sensitivity and specificity for individual HPV genotypes using a proficiency panel of HPV recombinant plasmids obtained in the context of the 4^th^ WHO HPV LabNet Proficiency Study for Evaluating HPV DNA Typing Methods. The system was evaluated as proficient for detection of HPV 16, 18, 31, 33, 35, 39, 45, 52, 56, 58, 59, 66, and 68b (http://www.who.int), being able to detect 50 genome equivalents (GE)/5 µL of HPV 16 and HPV 18 DNA, and 500 GE/5 μL of the other HPV genotypes with a specificity above 97%. Amplification products were subjected to electrophoresis on a 7% polyacrylamide gel followed by staining with ethidium bromide and image analysis by the Gel Doc imaging system (Bio-Rad Laboratories Inc., Hercules CA). HPV genotypes were identified by direct automated DNA sequencing analysis using both GP5+ and GP6+ primers for mucosal HPV and CP66, CP69 or CP68 primers for cutaneous HPV at Eurofins Genomics GmbH (Ebersberg, Germany). Subsequently, HPV genotypes were identified by alignment of HPV sequences with those present in the GenBank database using the BLASTn software (https://blast.ncbi.nlm.nih.gov/Blast.cgi).

### 2.4. Meta-Analysis

Published articles whose title, abstract or keywords referred to the detection of HPV DNA in newborns were searched in Medline using the terms (“Human Papillomavirus” OR “HPV”) AND (“infants” OR “newborns”) AND (“transmission”) (Figure S1). The search was updated on 23 January 2020.

The criteria for inclusion of the articles in the present meta-analysis were as follows: (1) explicitly provided information on HPV DNA detection method, such as PCR-based methods (i.e., real-time PCR or end-point PCR using broad-spectrum primers, HPV type-specific primers or a combination of both kinds of primers) or by non-PCR methods (i.e., chip array); (2) necessary data could be directly extracted or calculated from the original article. Articles containing duplicated data were integrated and review articles, studies in languages other than English as well as articles with low sensitive detection methods (before the year 2000) were excluded.

For each article included in the meta-analysis, sample types, percentages of HPV-positive samples, detected HPV types, delivery type and HPV detection methods were reported. All the data regarding newborns, their mothers and fathers (if available) were tabulated separately in a Microsoft Excel sheet (Table S2).

### 2.5. Statistical Analysis

The statistical analyses were performed using GraphPad Prism (version 6) software. The Χ^2^-test for trend was used for comparison of categorical data. Differences were considered statistically significant when *p* values were less than 0.05.

## 3. Results

Overall, HPV DNA sequences were detected in 14 out of 113 (12.4%) nasopharyngeal samples (Table 1). Beta HPV genotypes were found in 100% of HPV positive cases. No alpha HPV types were detected. The most common beta genotypes were HPV 20 Lancaster (21.4%) and HPV 107 (21.4%), followed by HPV 100 and RTRX7 (14.3%) (Table 2). The relative frequency of all other genotypes among positive samples was 7.1%. The higher prevalence of beta HPVs was observed among infants in the age group 2–6 months (16.9%), followed by those >6 months old (10%) and by the group 12 days–1 month old (5.3%). HPV 20 was the most frequent type in the 2–6 months’ age group (4.6%), followed by HPV 107 and HPV 100 (3%), RTRX7, HPV 5b, HPV 25 and HPV 152 (1.5%) (Table 2). HPV 107 and RTRX7 were detected in 50% of HPV positive infants aged 2 days–1 month (Table 2). No multiple HPV infections were detected. 

### Meta-Analysis

**Figure 1:**
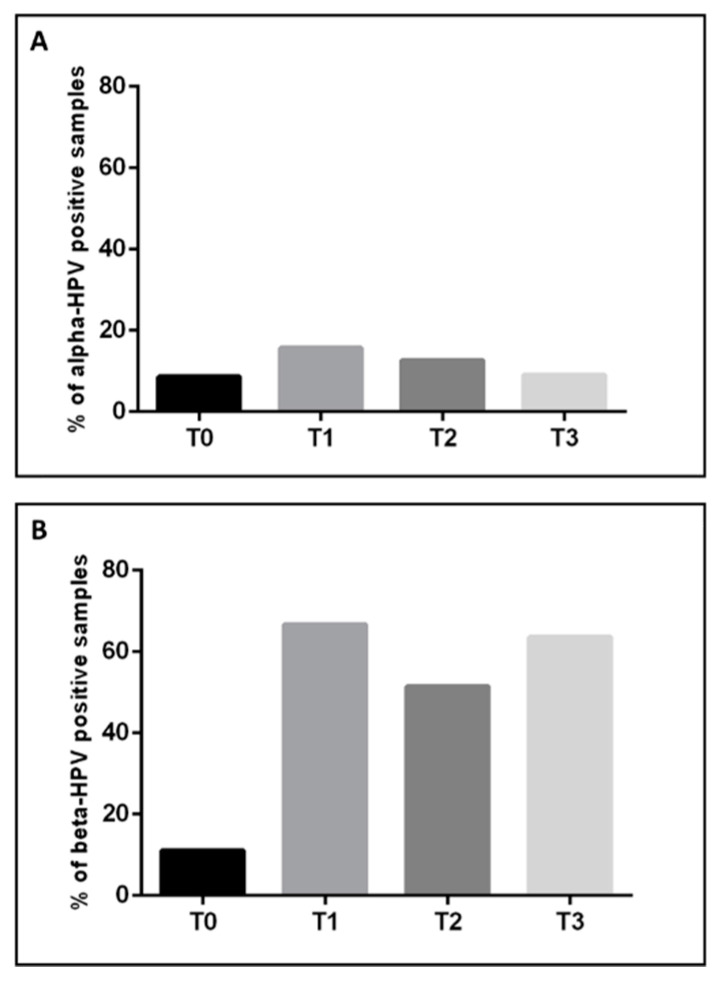
Frequencies of alpha (**A**) and beta (**B**) HPV samples in newborns at birth (T0), 1 day to 1 month old (T1), >1 month to 12 months old (T2) and >12 months old (T3).

A comprehensive analysis of published studies allowed us to evaluate HPV prevalence among 5126 newborns at different times from delivery. Specifically, alpha HPVs have been detected in 8.5% (282/3298) of samples obtained from infants at birth (T0), in 15.7% (108/689) of the 1 day–1 month old group (T1), in 12.7% (90/708) of >1 month–12 months old group (T2) and in 9.1% (7/77) in the group of >12 months old infants (T3). Beta HPVs have been detected in 11.1% (2/18) of samples from infants at birth (T0), in 66.7% (24/36) of the 1 day–1 month old group (T1), in 51.4% (18/35) of >1 month–12 months old group (T2) and in 63.6% (21/33) of infants >12 months old (T3), (Table 3), (Figure 1). Statistically significant negative and positive trends were found for alpha and beta HPVs, respectively, in T0 to T3 age groups (*p* < 0.001).

## 4. Discussion

HPV infection is known to have a sexual transmission, but other modalities of transmission have been described [11,32]. Several studies documented the transmission of HPVs from the mother to the child, with controversial results. In general, it is believed that the risk of viral transmission in newborns is associated with vaginal delivery [33].

In our study we selected breastfed infants born by vaginal delivery that sought medical care for respiratory illness and underwent a nasopharyngeal aspirate. Ten out of 113 infants were diagnosed with respiratory syncytial virus (RSV) by RT-PCR. The nasopharyngeal samples were also analyzed by using a broad-spectrum PCR designed for the detection of mucosal and cutaneous HPVs. None of the nasopharyngeal samples contained alpha HPV DNA sequences. Conversely, the rate of cutaneous HPV detection showed an age-dependent positive trend ranging from 5.3% to 16.9% in infants less than or more than 2 months old, respectively, and no association with RSV infection. We observed a decreasing rate of HPV positivity in the group of children older than 6 months (10%). The reason for such a decline may be due to multiple factors including the ability to contain infections following the maturation of children’s immune system or changes in the gut microbiota at weaning preventing pathogens growth.

Cutaneous HPVs all belonging to the beta genus included HPV 5b, HPV 20, HPV 25, HPV 100, HPV 107, HPV 124, RTRX7 and HPV 152. Particularly, the HPV 5b, HPV 20 and HPV 25 of the species beta 1 HPVs are possibly carcinogenic and frequently detected in cutaneous lesions [3,4,26,34]. The HPV 107, previously identified in cutaneous basal cell carcinoma (BCC), squamous cell carcinoma (SCC), actinic keratosis (AK) and seborrhoeic keratosis (SK) [35], is classified as species beta 2 having higher homology with HPV 9; the HPV 100 that we found in two samples in our study is highly homologous to HPV 22 and 23 [36]. The RTRX7, isolated in 1998 from an incipient squamous cell carcinoma of an immunosuppressed renal transplant recipient, is an EV-associated virus closer to HPV 12 (81% homology) [37]. The detection of HPV 107 as well as of several other beta HPVs in oral samples is possible due to their dual tropism for both skin tissues and mucosal epithelial sites [38]. Indeed, Forslund et al. detected HPV positivity in nasal (6%) and oral (50%) samples, with identification of beta HPVs in 4% of oral samples and in 31% of nasal specimens (prevalently HPV 24, 124, 76) [39]. Hence, there is still an open question about the tropism of some beta HPV types. 

It was reported that beta HPVs colonize the individual immediately after birth [40] and there is a quite strong agreement between those present in the mother and those detected in the child, by analyzing forehead swabs, the back of the right hand, and right side of the buttock swabs. In particular, in two pilot studies, two children tested negative at birth and, in the first case they were positive for HPV DNA after 1 week and for the following 8 weeks, and in the second case at 1, 3, 5, 8, 13, 21 days after birth [41]. Samples from children aged 1 month, 1 year and 4 years old tested positive for HPV DNA with a frequency of 60%, 50% and 70%, respectively, with a concordance rate, between HPV types found in the mother and in the child, of 25% (1 month), 40% (1 year), 0% (4 years). In total, 26 new putative HPV genotypes, 20 HPV genotypes and 27 putative HPV genotypes already characterized were discovered from the analysis of samples coming from the child, the mother and the environment, among which the most frequent were HPV 5, HPV 20, HPV 25 and RTRX7 [41].

The meta-analysis showed that detection of beta HPVs is less frequent at the birth and that it increases over the time (>12 months), as shown in Figure 1. Most of the studies that analyzed the vertical transmission of HPV have included different samples collected from the oral mucosa, from the upper respiratory airways, pharyngeal mucosa, the conjunctiva, nasopharyngeal aspirate as well as from cord blood and genital samples of children at birth and later times. However, the frequency of alpha HPV-positive samples has been shown to decrease rapidly after birth [33,42,43]. Hahn et al., for instance, observed that the frequency of alpha HPVs in mouth secretions and oral mucosa samples collected from newborns soon after delivery was 20.8% and all children became negative at the age of 2 months [33]. Rombaldi et al. also showed a decrease in the HPV positivity rate between the first and the sixth month of life in children who were born from HPV-positive mothers and were positive for HPV at birth, but became all negative for HPV after 12 months of life [16]. Alpha HPVs show a higher prevalence in vaginal/cervico-vaginal, oral, placental maternal samples, as well as in the peripheral blood of the mother, in the prepartum and in the peripartum period (Table S2). Two studies included in the meta-analysis [44,45] showed the presence of alpha HPV in breast milk during the postpartum period. Louvanto et al., reported no association between the HPV genotypes found in the mothers and those found in the oral mucosa of infants [44]; furthermore, Yoshida et al. did not find any concordance between HPV genotypes in the breast milk and those present in the newborns [12]. Conversely, Koskimaa et al., observed a significant concordance between the HPV genotypes suggestive of viral transmission from the mother’s epithelium to the oral cavity of the infant [45]. In one of the above mentioned studies [23] the most prevalent HPVs in nipple samples were genotypes 6 and 11, followed by others types such as HPV 16, 57, 27, 66, 37, 20, 21, 23, 32, 38. HPV 6 was identified in samples obtained from a child born to a mother with condylomata acuminata lesions [46]. The authors found also a considerable high titer of IgG antibodies reactive against HPV 6 either in the sera of mothers or of infants suggesting that neutralizing antibodies would be a useful tool to reduce the risk of HPV-related diseases in the newborns. 

Various factors may contribute to the risk of HPV transmission, such as the presence of cervical warts in the mother (condylomata acuminata, cervical dysplasia, cervical cancer) [17], maternal age and history of immunosuppression (e.g., HIV) [47,48]. Nevertheless, some children tested positive for HPV DNA although apparently their parents did not have a HPV-related disease. A possible intrauterine transmission through leakage of amniotic fluid or via placental infection has been also described [32]. Infants born from mothers with cervical exfoliates testing negative for the virus usually are negative for HPV, while those born from mothers with cervical HPV infections are frequently positive with an elevated viral genotype concordance between mother and child [16,49]. However, a study conducted at the University of Iowa Hospitals and Clinics showed that genotype concordance in child/mother couples was observed only in 1 on 3 children born from HPV-positive mothers [50]. Furthermore, in a prospective Finnish HPV family study [51], high risk HPV DNA detection showed a decreasing rate of carriage during the first year of life while it was still detectable in only 10% of infants during the 3 years of follow-up. The higher rate of HPV detection in older children as well as the ambiguous concordance between HPV types in mother and child suggests that HPV could be transferred by other routes including contacts with untested relatives [52]. In addition, a study performed in the Spanish population showed a rate of 58% (53/91 neonates) HPV positivity in neonatal oropharyngeal specimens at birth and 30% (27/91) positivity in the seven days after the partum with a clearance of 49% (26/53) [49]. Another study reported an HPV detection frequency of 51.6% in oral samples from Spanish newborns [53].

A limitation of the present study is represented by the relatively small sample size. However, the use of broad-spectrum PCR and direct sequence analyses allowed us to identify uncommon HPV genotypes partially different from those described in the literature until now. Since the samples were collected quite far from birth, we may suppose that infection has occurred postnatally. Hence, the prevalence of HPV 6 and 11 is shown to be strongly predictive of a JoRRP and the presence of condylomata acuminata in women, caused by HPV 6 and 11, is one of the factors involved in the onset of RRP in the infants; we wonder about the role of the uncommon viral genotypes (i.e., HPV107) isolated in our study. So, we ask if they could cause some type of disease either in younger or in adult individuals. Recently, it was shown that the presence of some beta HPV types in the oral cavity is associated with an increased risk to develop head and neck cancer [54]. It is of fundamental importance, in this scenario, to understand if viral DNA of beta HPVs has a pathogenic role in infants or if it is only a passenger infection without the possibility to cause an actual disease. 

## Figures and Tables

**Table 1:** Baseline information of the 113 children included in the study and human papillomavirus (HPV) detection results.

	Patient Variability	Cases *n*	HPV Negative *n* (%)	HPV Positive *n* (%)	Alpha HPV *n* (%)	Beta HPV *n* (%)
Age	<2 months (T1)	38	36 (94.7%)	2 (5.3%)	0 (0%)	2 (5.3%)
	2–6 months (T2)	65	54 (83.1%)	11 (16.9%)	0 (0%)	11 (16.9%)
	>6 months (T3)	10	9 (90%)	1 (10%)	0 (0%)	1 (10%)
	**TOT**	**113**	**99 (87.6%)**	**14 (12.4%)**	**0 (0%)**	**14 (12.4%)**
Sex	Male	61	56 (91.8%)	5 (8.2%)	0 (0%)	5 (8.2%)
	Female	52	43 (82.7%)	9 (17.3%)	0 (0%)	9 (17.3%)

**Table 2:** List of HPV types detected in the study, genus and species, and relative percentage among children.

Beta HPV Genotype (Genus, Species)	Cases *n* = 14 (%)	T1 (<2 m) *n* = 38 (%)	T2 (2 m–6 m) *n* = 65 (%)	T3 (>6 m) *n* = 10 (%)
HPV 20 (β,1)	3 (21.4%)	0 (0%)	3 (4.6%)	0 (0%)
HPV 107 (β,2)	3 (21.4%)	1 (2.6%)	2 (3%)	0 (0%)
HPV 100 (β,2)	2 (14.3%)	0 (0%)	2 (3%)	0 (0%)
RTRX7 (β,1)	2 (14.3%)	1 (2.6%)	1 (1.5%)	0 (0%)
HPV 124 (β,1)	1 (7.1%)	0 (0%)	0 (0%)	1 (10%)
HPV 25 (β,1)	1 (7.1%)	0 (0%)	1 (1.5%)	0 (0%)
HPV 5b (β,1)	1 (7.1%)	0 (0%)	1 (1.5%)	0 (0%)
HPV 152 (β,1)	1 (7.1%)	0 (0%)	1 (1.5%)	0 (0%)
TOT	14 (12.4%)	2 (5.3%)	11 (16.9%)	1 (10%)

**Table 3:** Prevalence of alpha and beta HPVs in nasopharyngeal samples of infants at and after birth.

	Alpha HPV		Beta HPV	
Age	HPV + /Total	%	HPV + /Total	%
**T0: at birth**	282/3298	8.5%	2/18	11.1%
**T1: 1–30 days**	108/689	15.7%	24/36	66.7%
**T2: >1–12 months**	90/708	12.7%	18/35	51.4%
**T3: >12 months**	7/77	9.1%	21/33	63.6%

The Χ^2^ test for trend was statistically significant for alpha (negative trend) and beta (positive trend) HPVs (*p* < 0.001).

## Supplementary Materials

The following are available online at https://www.mdpi.com/1999-4915/12/10/1119/s1, Figure S1: Flow diagram of selected articles and inclusion criteria of the meta-analysis; Table S1: list of oligonucleotide primers used and relative name, sequence, and PCR conditions; Table S2: Microsoft Excel sheet shows all the data regarding newborns, their mothers and fathers collected for the meta-analysis.
